# Pneumocephalus Secondary to Cerebral Air Embolism After Acute Bleeding in an Emphysema Bulla

**DOI:** 10.7759/cureus.39051

**Published:** 2023-05-15

**Authors:** Sofia Dinis-Ferreira, Margarida Jardim, Duarte Freitas, José J Nóbrega

**Affiliations:** 1 Intensive Care Unit, Hospital Central do Funchal, Funchal, PRT; 2 Radiology, Hospital Particular da Madeira, Funchal, PRT; 3 Intensive Care Department, Hospital Central do Funchal, Funchal, PRT

**Keywords:** intensive care, hyperbaric oxygen therapy (hbot), pneumocephalus, emphysema, cerebral air embolism

## Abstract

Pneumocephalus is the presence of air in the intracranial space and has multiple causes, including cerebral air embolism. Its presentation may range from asymptomatic to decrease mental status, coma, and seizures.

We present a case of cerebral air embolism secondary to acute bleeding inside an emphysema bulla. A 69-year-old female was brought to the emergency room after suffering acute dyspnea, convulsions, and cardiac arrest during a commercial flight. The Head CT showed the presence of multiple small gas collections in the brain, and the Thoracic Angiotomography showed a thin-walled bulla surrounded with pulmonary venous vascular structures and signs of active bleeding. The patient had rapid neurological deterioration with evolution to brain death due to anoxic encephalopathy before the possibility of treatment with pulmonary lobectomy and hyperbaric oxygen therapy.

It is important to identify the localization of pneumocephalus to determine its etiology and to deliver the best treatment. Cerebral air embolism may happen when air enters the arterial or venous system, which can cause brain damage due to capillary leak syndrome and local ischemia. Treatment of pneumocephalus includes treating the cause, bed rest, avoidance of Valsalva maneuvers, positive pressure, and hyperbaric oxygen therapy. Early recognition is essential to prevent complications such as irreversible brain lesions and to improve patient outcomes.

## Introduction

Pneumocephalus refers to the presence of air in the intracranial space and can happen for several reasons, including trauma, iatrogeny from cranial procedures, air entry after device placement, and infections [1]. Patients may be asymptomatic, however, some may present with decreased mental status, focal signs, and seizures depending on the volume and rate of air introduced [2]. The main treatment includes interruption of further air entry, supportive care, administration of high oxygen concentrations, and hyperbaric oxygen therapy (HBOT) [2]. We report a case of pneumocephalus after acute bleeding inside an emphysema bulla with secondary cerebral air embolism.

## Case presentation

A 69-years-old patient was brought to the hospital after suffering sudden dyspnea, seizures, and cardiac arrest during a 3-hour commercial flight, approximately 20 minutes after take-off. The patient had a history of arterial hypertension and emphysema with no history of smoking, recent surgeries, or diving activities. She was previously asymptomatic. Promptly assistance was provided on board with the flight attendants and a medical doctor, cardiopulmonary life support maneuvers were initiated, and return of spontaneous circulation was documented after 10 minutes. After the plane landed, the pre-hospital emergency team noticed the patient was cyanotic and with a left conjugate eye deviation. She was intubated and ventilated after sedation with propofol and was brought to the hospital. On arrival at the emergency room, she had a Glasgow Coma Scale of 3, and pupils were on the midline, isochoric, and equally reactive to light. She was ventilated in volume-controlled mode with a fraction of inspired oxygen at 1.0. The blood gas analysis was consistent with respiratory acidosis and severe hypoxemia (pH 7,16, pCO_2_ 80.8 mmHg, pO_2_ 98.3 mmHg, P/F 98.3, HCO_3_ 25 mmol/L). There was no thoracic asymmetry, tracheal deviation, or alterations in pulmonary auscultation. She was under vasopressor support with noradrenaline at 0.4 mcg/Kg/min for Mean Arterial Pressure of 65mmHg, capillary refill time was 2 seconds, and lactate was 2.8 mmol/L. The electrocardiogram showed no alterations. She had a capillary blood glucose of 288 mg/dL and a hypokalemia of 2.8 mEq/L. Ventilatory adjustments were made to improve the respiratory acidosis, 1 gram of Levetiracetam was administered, and potassium replacement was started. A Head CT and a Thoracic Computed tomography angiography (CTA) were performed.

**Figure 1:**
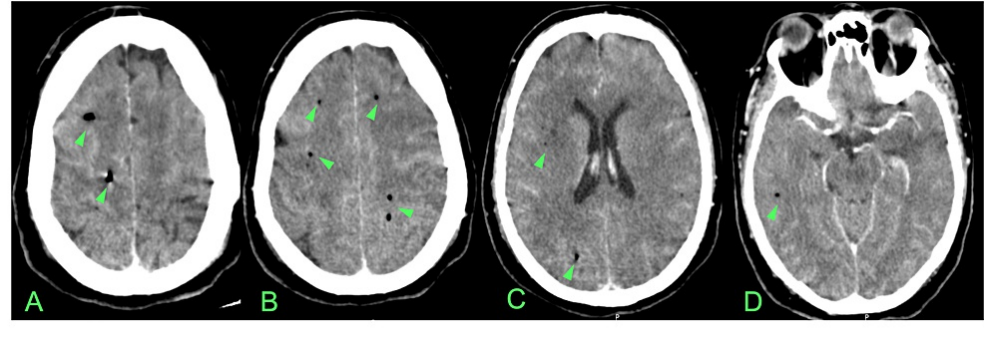
Head CT scan at admission with various axial cuts (A-D) revealing multiple small gas collections dispersed in both cerebral hemispheres (arrowheads).

**Figure 2:**
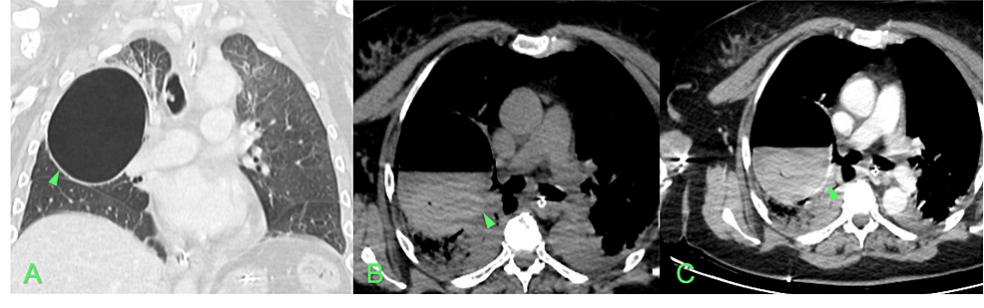
Thoracic CTA at admission with coronal cut (A) showing the emphysema bullae (arrowhead). In the axial cut, the right lung lobe is filled with heterogeneous fluid before (B) and after (C) contrast administration (arrowhead).

The Head CT showed the presence of multiple small gas collections dispersed in both cerebral hemispheres and some cerebral edema, with no cranial bone alterations (Figure 1). The Thoracic CTA ruled out pulmonary embolism, however, it showed a thin-walled bulla measuring 8.8x8.5 cm in the right lung lobe with heterogeneous air-fluid level with signs of active bleeding into the lumen after contrast administration (Figure 2). Also, there was parenchymal densification in the upper lobes, apical and posterior segments of the lower lobes consistent with nonspecific alveolar filling.

**Figure 3:**
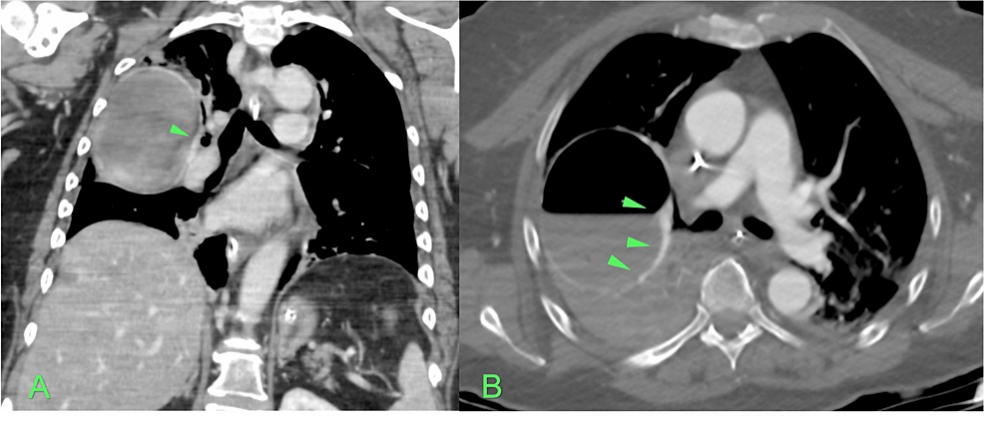
Thoracic CTA with coronal (A) and axial (B) cut of the bulla surrounded by pulmonary venous vascular structures (green arrows)

**Figure 4:**
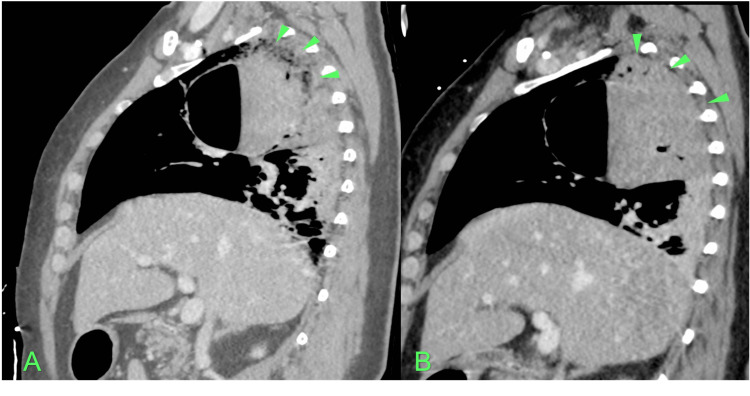
Sagittal plane of the first (A) and second (B) Thoracic CTA where’s noted an increase in the densification of the adjacent pulmonary parenchyma compatible with hematic alveolar filling (Green arrows).

The patient was admitted to the Intensive Care Unit for further stabilization. An echocardiogram was performed and found to be within the normal range for age, ruling out the presence of interatrial and interventricular communication. A new Thoracic CTA with vessel reconstruction was requested to try to identify the bleeding vessel. It was not possible to identify active extravasation of contrast compatible with active hemorrhage, however, it was noted that this bulla was surrounded by pulmonary venous vascular structures (Figure 3). It also noticed a slight increase in the densification of the adjacent pulmonary parenchyma compatible with hematic alveolar filling (Figure 4). It was not possible to exclude intravascular gas.

**Figure 5:**
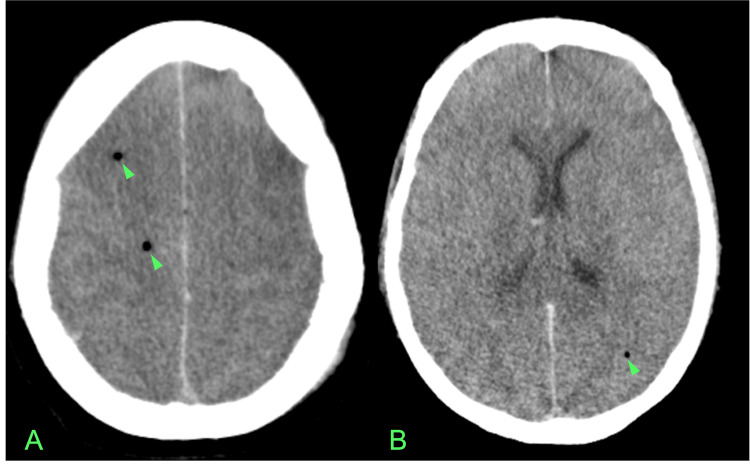
Head CT-scan after 12 hours of admission showing loss of cortico-subcortical differentiation (A), as well as effacement of the basal ganglia (B) compatible with diffuse cerebral edema. Only three small gas collections were found (arrowheads).

After these findings, the case was discussed with Cardiology and Cardiothoracic Surgery, and the patient was considered for surgical treatment with right upper lobectomy, and HBOT was equated. However, her neurological condition quickly deteriorated with bilateral mydriasis and the absence of brainstem reflexes after 12 hours of admission. A new head CT scan showed diffuse cerebral edema (Figure 5) and anoxic encephalopathy. The patient was formally evaluated, and brain death was declared after three days after admission.

## Discussion

When identifying pneumocephalus, it's important to localize the gas to a compartment (intravascular, intraparenchymal, subdural, epidural, subarachnoid, and intraventricular) and to determine its route of entry (through the skull, extension from a paranasal sinus or the mastoid air cells, via the spine, or trans-vascular), since it helps to determine the etiology [3].

Cerebral air embolism happens through the arterial system when air enters the brain during operative or interventional procedures, through right-to-left shunting in the heart or lungs (foramen oval, atrial septal defects, or pulmonary arteriovenous fistula) or introduced directly into the pulmonary vein during a lung intervention [4,5]. Also, air may enter the pulmonary venous system when a direct connection with the atmosphere is created through a bronchial venous fistula or the pulmonary artery, then pass to the pulmonary capillary bed and then to the pulmonary venous system [4,5]. Cerebral air embolism causes edema secondary to capillary leak syndrome and local ischemia due to embolism, with multifocal lesions resulting in various neurological deficits [6].

According to Boyle's law, the volume of gas is inversely proportionate to the pressure, hence the risk of air expansion during the ascending part of air traveling, which may cause hypoxemia, barotrauma, acute bleeding, and air embolism, particularly in patients with a previous history of emphysema bullae and chronic obstructive lung disease. [7]. In our case report, gas may have been introduced directly into the pulmonary veins surrounding the emphysema bullae after the acute bleeding, then traveled to the left auricle and left ventricle, and then embolized the brain, which is consistent with the presence of gas in multiple intracranial locations. As previously mentioned, there was no history of recent surgical intervention nor invasive device placement and no diving incidents. This is an extremely rare cause of air embolism, and only a few cases are described in the literature [8-13]. In all of them, including with our patient, symptoms occurred during the ascending part of the flight and included hypoxemia, hemoptysis, loss of consciousness, and convulsions. Two cases were fatal [12,13]

Treatment of pneumocephalus includes non-pharmacological measures such as bed rest, 30º degree head position, high flow oxygen, and avoidance of Valsalva maneuvers and positive pressure [14]. HBOT is indicated in the presence of neurological deficits [15]. It works by promoting gas reabsorption, reducing ischemic injuries, and improving tissue oxygenation [15]. Early HBOT in the first 6 hours provides a better outcome with less sequelae and death rate, but there's also evidence of late benefits of its use up to 60 hours after the event onset [16]. However, HBOT may be contraindicated in patients with underlying respiratory pathologies since it could increase the risk of barotrauma [16]. Unfortunately, our patient already had cerebral edema on admission head CT scan and showed rapid clinical deterioration before being able to carry out the proposed treatment.

## Conclusions

Pneumocephalus may have several causes, and determining its etiology is essential for managing the patient to avoid serious complications. Cerebral vascular air embolism should be excluded in the presence of acute neurologic symptoms when an entry of air into the arterial or venous system is suspected. Definitive treatment includes stopping the cause of the embolism, high concentrations of oxygen, and hyperbaric oxygen therapy. Patients with emphysema bullae should be evaluated before flying for risk assessment and implementation of preventive measures.
